# Moving towards less biased research

**DOI:** 10.1136/bmjos-2020-100116

**Published:** 2021-01-17

**Authors:** Mark Yarborough

**Affiliations:** Bioethics Program, University of California Davis, Sacramento, California, USA

**Keywords:** bias, research ethics, conflict of interest

## Introduction

Bias, perhaps best described as ‘any process at any stage of inference which tends to produce results or conclusions that differ systematically from the truth,’ can pollute the entire spectrum of research, including its design, analysis, interpretation and reporting.^1^ It can taint entire bodies of research as much as it can individual studies.^2 3^ Given this extensive detrimental impact, effective efforts to combat bias are critically important to biomedical research’s goal of improving healthcare. Champions for such efforts can currently be found among individual investigators, journals, research sponsors and research regulators. The central focus of this essay is assessing the effectiveness of some of the efforts currently being championed and proposing new ones.

Current efforts fall mainly into two domains, one meant to prevent bias and one meant to detect it. Much like a proverbial chain, efforts in either domain are hampered by their weakest components. Hence, it behoves us to constantly probe antibias tools so that we can identify weak components and seek ways to compensate for them. Further, given the high stakes—conclusions that align with rather than diverge from truth—it further behoves the biomedical research community to prioritise to the extent possible bias prevention over bias detection. The less likely any given study is to be tainted by bias, the fewer research publications reporting biased results there will be. The value of detected bias pales in comparison, for it extends only as far as those who are aware of that detection after the fact, meaning that biased conclusions at variance with the truth can mislead those unaware of the bias that taints them for as long as the affected publications endure.

With these preliminary considerations about bias in mind, let us first examine some current antibias efforts and probe their weaknesses. Doing so will show why we need to develop additional strategies for preventing bias in the first place, and space is set aside at the end to examine two related candidate strategies for how we could attempt to do that.

## Current bias countermeasures

Table 1 reflects some current countermeasures being employed to combat various kinds of biases. Though the table is far from comprehensive, (dozens of biases have been catalogued)^1^ it does include major biases of concern, representative countermeasures to combat them, whether those countermeasures prevent or detect bias, and their likely relative strength.

**Table 1:** 

Bias example	Examples of harm resulting from bias	Current prevalent bias countermeasures	Countermeasures goal	Likely strength of the current bias countermeasures
Sponsorship bias^4^	Possible suppression of critical evidence	Disclosure of financial relationship	Bias Detection	Weak^7 13 14 18 50 51^
Selection, performance and detection biases^52^	Publications that report what are likely to be false positive findings	Peer Review Journal Checklists Investigator Guidelines	Bias Detection and Bias Prevention	Variable^53 54^ Variable^35 36 55 56^ Variable^52^
Publication biases (eg, selective reporting and non-reporting of outcomes)	Inaccurate and/or irreproducible findings	Peer review Reporting bias tools for use in systematic reviews (eg, scales and checklists)^57 58^	Bias Detection	Variable^2 32^ Variable to Weak^59–62^

### Sponsorship bias

The bias that probably draws the most attention is what is known as sponsorship bias,^4 5^ wherein pecuniary interests undermine the disinterestedness meant to prevail in scientific investigations.^6^ The most prominent countermeasure against it consists in multiple disclosure practices that flag financial relationships between scientists and private companies. For example, academic institutions may require faculty to disclose annually their financial relationships with private companies; research sponsors may require applicants to make such disclosures when submitting applications; and journals typically require authors to make such disclosures when submitting manuscripts. The right-hand column of table 1 prompts the question, ‘to what extent do such disclosures actually prevent sponsorship bias?’ There is now ample conceptual analysis^7–10^ and empirical evidence produced over many years such that we can safely state that there is an over-reliance on disclosure.

This extensive prior work shows, for example, that journal disclosure policies targeting authors fail to capture many financial ties between researchers and industry. Recent studies show that consulting agreements between researchers and companies, as well as financial ties between biomedical companies and organisations that produce clinical practice guidelines, often go undisclosed.^11 12^ Looking at journal disclosure policies, we see further evidence of disclosure’s limited ameliorative effect. A recent study that randomised article reviewers into one group that received financial interests disclosures along with the manuscripts to be reviewed and another group that did not found that the disclosures had no effect on reviewer assessments of the manuscripts.^13^ Another recent study looked at editorial practices regarding the financial interests of authors at 30 leading medical journals and found that none had actual tools for determining whether and how disclosed financial relationships might have impacted any given research report.^14^

Additional considerations help to further explain the weaknesses of journal disclosure policies. First, disclosures are usually mistimed. When financial relationships bias studies, that bias occurs long before anyone discloses the relationships in reports about the studies.^15^ Second, it is those, and only those, designated as authors who are subject to them. Often those who lead the design, conduct, analysis and reporting of a study are not in fact considered authors of it.^16^ Private companies that sponsor the majority of drug studies and/or contract research organisations they hire control the design, manage the conduct, and analyse the data, as well as write the articles about that analysis for studies.^17^ Journal disclosure mandates leave untouched the bias that these conflicted sponsors can introduce into clinical trials because of sizeable holes in the International Committee of Medical Journal Editors (ICMJE) authorship policy. Followed by an outsized portion of biomedical research journals, it ‘support[s] practices of commercial data control, content development and attribution that run counter to science’s values of openness, objectivity and truthfulness’ because ‘the ICMJE accepts the use of commercial editorial teams to produce manuscripts, which is a potential source of bias, and accepts private company ownership and analysis of clinical trial data.’^16^ In other words, even though readers of journals assume that journals accurately attribute those, and only those, who are responsible for the design, conduct, analysis and reporting of a study, authorship practices do not in fact require such accurate attribution. Thus, we are relying on disclosure, often after the fact of conducting a study, to combat the bias that financial entanglements can cause prior to a study’s launch and the disclosure practices themselves often mistarget those who should be making the disclosures. The end result is that current disclosure practices can conceal rather than reveal the prospect of sponsorship bias.

Furthermore, even if disclosures were better targeted, this would not negate the potential that disclosures themselves have to cause unintended detrimental consequences. Commentators long ago noted that disclosing financial relationships may contribute to people having a sense of ‘moral license to (act in biased ways more) than they would without disclosure. With disclosure, (acting in a biased way) might seem like fair play. While most professionals might care about their (audience), disclosure (practices) can encourage these professionals to exhibit this concern in a merely perfunctory way.’^18^

There are two final considerations about disclosure that need to be noted. First, disclosure is not meant to actually detect bias. Rather, it is meant to alert people to its possibility. Thus, even though disclosure is our major tool for combating one of the most detrimental forms of bias, it is not clear what good it actually does, which leads us to the second consideration. Since disclosure does nothing to prevent sponsorship bias, more substantial countermeasures aimed at prevention are needed. It is beyond the scope of this essay to examine the suitability of possible countermeasures for preventing sponsorship bias, such as sequestering investigators from private companies whenever possible.^15^ Referencing this one example, though, highlights the substantial difference there can be between detecting bias on the one hand and actually preventing it on the other, a topic we will return to later.

Returning for the moment, though, to detection of sponsorship bias, these collective concerns about the most prevalent safeguard against it suggest that it can facilitate rather than detect, let alone prevent, bias. By stopping at disclosure, it suggests that financial entanglements are often permissible; we just need to make sure they are relatively transparent to others. The end result is that there is a pall of uncertainty cast over a large body of published research, including a major portion of the clinical trials that society relies on to improve healthcare.^17^

### Additional major sources of bias

Evidence about the effectiveness of safeguards against other prominent sources of bias besides sponsorship bias is equally disconcerting. Consider, for example, biases that impact the design, conduct and reporting of preclinical animal studies. This class of studies is of particular concern for multiple reasons, not the least of which is the fact that early phase clinical trials, and the risks intrinsic to them, can launch on a single, highly prized ‘proof-of-concept finding in an animal model without wider preclinical validation.’^19^ This risk is particularly grave when we consider the interests and welfare of the patients who volunteer for the early phase clinical trials.^20^

Given such high stakes, it is critical that there be effective safeguards that, once again, counter biases that undermine the rigour that studies capable of producing reliable findings require. Here too table 1 prompts investigation of how well current safeguards actually work. Evidence about excess significance bias, a publishing bias due in large part to selective publishing of results by both authors and journals, shows major limitations in their effectiveness. Looking, for example, at the neurosciences preclinical studies generally^2^ and stroke studies specifically,^21^ we see that excess significance bias is a major contributor to well documented failure^22 23^ to successfully ‘translate preclinical animal research (results) to clinical trials.’^24^

When we look at biases resulting from poor study design, across all fields of preclinical inquiry, we find that studies that lack construct, internal and/or external validity that produce biased research reports are ubiquitous.^25^ Not only have such findings contributed to ‘spectacular failures of irreproducibility’^25^ that cast concern over entire fields of research,^3^ they also forecast failure for the clinical trials that seek to translate preclinical findings into clinical therapies.^26^ Illustrating this is a recent study estimating that a majority of the reports of positive findings from animal studies meant to inform clinical studies of acute stroke actually report what are likely to be false positive results.^27^

With this evidence in mind, we must consider anew the harm caused by, for example, toxicities, personal expenses and opportunity costs^28^ that phase 1 trial participants endure in trials that launch on the basis of preclinical studies whose biased design produces unreliable research reports used to justify the clinical trials.^29^ Those participants have no choice but to rely on a properly functioning research oversight system to protect their interests and welfare. Alas, that oversight system is much weaker than the research and research oversight communities likely would care to admit.^30^ All the more reason, then, that our efforts to guard against bias should be as varied and robust as its many sources.

The fact of the matter, though, is that the most prominent safeguard against them is peer review. Since it occurs at the reporting stage of the research continuum, it is preceded by other safeguards, such as reporting guidelines, which are reviewed below. None of these other safeguards are as ubiquitous as peer review, however, and it is the gate that publications must ultimately navigate through. Given this level of significance, its effectiveness warrants careful scrutiny. Scrutiny begins by noting that peer review is meant to detect rather than prevent bias. One perhaps could counter that peer review actually is a hybrid countermeasure since it is capable of actually preventing bias at times, or at the least the dissemination of reports tainted by it since, when peer review works, it can prevent publication of suspect findings. However, though it is no doubt true that peer reviewers can reject manuscripts out of concern for bias, concerns about false positive findings, and the like, there is no assurance that manuscripts rejected at one journal will be rejected by all journals. Hence, even if one were to confer it a hybrid status wherein it can both prevent and detect bias, the extent of bias that has long been documented in peer-reviewed journals reveals major weaknesses in peer review. Recent high-profile COVID-19 -related retractions^31^ and commentary^32^ further confirms these weaknesses. Consequently, we need to be guarded in our expectations about the central antibias safeguard and its ability to assure the reliability of published research findings.

The upshot of all this is that current bias safeguards do little to alert clinical investigators, research ethics review committees, and others to the prospects of biased findings in either pivotal preclinical studies that are the precursors to clinical trials or the full spectrum of clinical trials themselves. This raises genuine concerns that far too many ill-advised clinical trials get conducted rather than avoided. It also underscores the need for conducting the individual studies that constitute any given body of preclinical or clinical research in a manner that is free of bias in the first place. Additional safeguards that prevent rather than detect bias will be needed if we are to succeed at this. No doubt multiple ones are needed. In the balance of this piece, I will focus on ones that could be used for preclinical studies, leaving clinical studies safeguards for other occasions.

## Preventing bias

### Examples of current bias prevention tools

We are fortunate that there are some safeguards for combatting bias in preclinical studies already in place. Perhaps the most notable are reporting guidelines such as the ARRIVE (Animal Research: Reporting of In Vivo Experiments) guidelines.^33^ Recently revised,^34^ the guidelines are designed to assure transparency of critical methodological aspects of animal studies. If widely enough adopted, they should promote greater rigour in animal research and thus prevent much of the bias that currently plagues it. Unfortunately, though, uptake of the guidelines has been lacklustre to date, mainly because too many animal researchers are either unaware of them or do not follow them.^35^ Not all the evidence about reporting guidelines is so discouraging though. A recent study of reporting guidelines tailored for the journal *Stroke* found that they substantially improved the quality of published preclinical studies when compared with reports in other journals that did not require use of the same guidelines.^36 37^

Despite the mixed evidence about the effectiveness of reporting guidelines, both general and journal-tailored reporting guidelines do have value that is worth noting. Even though they target the reporting stage of research, their use can influence how researchers design and conduct their studies. This highlights the true promise of reporting guidelines: they can incline researchers toward well-designed research and robust reports about it. To the extent that this occurs, they function as true bias prevention safeguards.

Nevertheless, enthusiasm for reporting guidelines must be tempered by the mixed evidence about them to date. It suggests that reporting guidelines will have an incremental effect at best on preventing bias. This is borne out by evidence, for example, pertaining to the TREAT-NMD Advisory Committee for Therapeutics. Although this committee does not promulgate specific reporting guidelines, it does promote the kinds of research practices that reporting guidelines are meant to foster. It does this by ‘provid(ing) detailed constructive feedback on clinical proposals for neuromuscular diseases submitted by researchers in both academia and industry.’ This group provided feedback on just under 60 preclinical research programmes between 2010 and 2019. It reports having raised concerns in just under a third of their reviews about the use of control groups, blinding and randomisation with researchers whose preclinical research they reviewed. They also report raising concerns about a misalignment between preclinical data and claimed preclinical efficacy almost a third of the time as well.^19^ While some may take comfort in the fact that the group’s reviews found deficiencies in basic elements of sound research in far less than half of the studies they reviewed, all likely agree that the frequency of deficiencies still remains troubling.

### Two new strategies for preventing bias in preclinical studies

Experience with the ARRIVE guidelines to date suggest that systematic adoption of new research practices will be sporadic, though, rather than widespread until we find ways to systematically move towards widespread adoption of reforms aimed at preventing bias. Perhaps the first step in moving in that direction is collectively grappling with an obvious inference to be drawn from all the evidence noted above: current success metrics in research can too often reward rather than prevent biased research. People may enjoy rewards from design-deficient studies, in the form of publications and funding, as well as the prestige that follows both. This suggests that efforts to combat bias are not just hampered by ineffective and often ill-timed bias countermeasures. They are also hampered by current flawed and entrenched incentive structures and researcher performance metrics that Hardwicke and Ioannidis contend ‘preferentially valu[e] aesthetics over authenticity.’^38^ While many readers may not agree that the current incentive structures are this far askew, we nevertheless must worry, based on the assembled evidence, that research institutions and sponsors may often incentivise biases in very much the same way that private sponsors can cause sponsorship bias.

If this analysis is sound, then widespread adoption of research practices capable of preventing bias will hinge on resisting current incentive structures. The most logical opportunity for generating such resistance resides jointly, I think, with institutional leaders and individual investigators. Though systems-level incentive structures contribute to biased research, the fact of the matter is that investigative teams conduct research and their members are trained at and often employed by research institutions. Thus, the path forward seems to depend on finding ways to get both investigators and research institutions to prize ‘authenticity’ more. This, no doubt, will prove challenging given the extent to which both groups can flourish under current rewards structures.

There are at least two complimentary strategies to look at that might prove beneficial. One encourages both investigators and research institutions to recognise the extent to which they are entangled in a major conflict of interest. Their primary interest in conducting authentic science is too often at odds with the secondary interest in being successful and enjoying the individual and institutional rewards of that success. Though we typically do not label this situation as a conflict of interest, often preferring instead the nomenclature of conflicts of commitment, the situation most assuredly is just as deeply conflicted as are the financial relationships that create sponsorship bias. If it was so designated, continued indifference about it would be difficult to maintain. That prospect alone warrants us labelling the situation the conflict of interest that it is.

The other strategy might provide additional motivation. It requires research teams and research institutions, either separately or jointly, to carefully examine the extent to which they may be contributing to the production of biased research. Here is one way they could do that: identify a systematic review of a given body of research in a given field that those participating in the exercise agree employed a reliable meta-analysis plan that identified bias and/or research deficiencies, determine whether any of the published studies included in the review originated from one’s lab or institution, and determine whether that study may have been at risk for contributing to the bias/deficiencies reported in the systematic review. If no studies from a lab group or the institution were included in the systematic review, they could still determine whether there are any published studies from the lab or institution that could have been included in the systematic review and, if so, whether their studies would have contributed to the worrisome findings reported in the systematic review. With these results in hand, the next step would be to develop a prevention plan that is designed to prevent future studies from exhibiting those problems. With the prevention plan in place, one could then determine what institutional and/or lab-level changes would be required in order to implement the prevention plan.

It is likely that few, if any, prevention plans would need to start from scratch. As most readers of this journal are no doubt aware, there is already a wealth of published scholarship about how to improve the quality of biomedical research. Some of the most relevant examples from it include routine use of study preregistration^39 40^ and research reports,^38 41 42^ supplementing the 3Rs^43^ of animal studies with the 3 Vs of scientific validity,^25^ and clearly reporting whether a study is a hypothesis generating or a hypothesis confirming study.^26^

We must acknowledge at the outset, though, that developing a prevention plan will likely prove much easier than fully adopting one because adoption will reveal how deeply entrenched the conflict of interest between professional success and rewards and good science often is. For example, clearly labelling research studies as exploratory ones in publications will temper claims about innovation that researchers may be accustomed to making about their work. Similarly, employing research reports will restrict study analyses and descriptions, which will often result in more constrained publications.^41^ Different researchers no doubt will respond differently to these changes, but one can hope that enough of them will feel empowered by the changes to become champions of science reforms within their institutions and professional societies meant to align success metrics with good research. Supporting this expectation are recent studies reporting that researchers are eager for improved research climates at their organisations.^44 45^

While research teams develop and implement prevention plans, institutional leaders will need to take responsibility for eliminating the conflicts of interest that promote bias in research. They would not need to start from scratch either, since important preliminary work that could help with this is already underway. This work includes efforts that show how to align institutional metrics of professional success with good science.^46–48^ An additional resource they could fruitfully draw from is the recently published ‘Hong Kong Principles for assessing researchers.’^49^ Here too it will no doubt be easier to develop than implement plans meant to avoid the entrenched conflict of interest. But benefits may quickly materialise as soon as the work to develop prevention plans materialise. Once institutions name, and thus acknowledge, the conflict of interest that they are helping to perpetuate, maintaining the status quo should prove that much more difficult. This should help to create at least some inertia tilted toward reform and thus away from stasis.

Many readers will no doubt be less sanguine about the success prospects for either strategy. The teams and institutions that choose to adopt them would no doubt have concerns that they would be unilaterally placing themselves at a disadvantage to those that choose not to burden themselves with the demands of either of the proposed strategies. With such concerns in mind, it is helpful to ponder how we might address them. Probably the best option for doing so is to implement some pilot projects to test the use of systematic reviews to develop bias prevention plans. There are at least two options for implementing such pilot projects.

One is for either an institution or a professional society to host a competition where the team that develops the best prevention plan for their work receives some kind of institutional/professional society recognition or reward. Institutional rewards might be monetary in the form of travel stipends for graduate students or postdoctoral fellows to attend conferences. Professional society rewards might be a plenary session at a society’s annual meeting where the winning team could present its bias prevention plan.

The other option is for research institutions to work through their main research officers to sponsor audits of the work of research teams. The audits would be informed by relevant systematic reviews. The audits could either be random or limited to teams that volunteer. To ensure that the audits are not seen or experienced as punitive, the launch of the audits would need to be preceded by a communication campaign that explained the purpose and value of the audits. Others may identify additional options for implementing pilot projects. Whatever options research teams, institutions, and/or professional societies might use, such pilot projects should prove valuable. They are likely the quickest way to learn whether systematic reviews could be used to interrogate research quality at the local level and to develop prevention plans for reducing bias in research.

## Conclusion

There is no one panacea capable of turning away all the contributors to decades of disappointing clinical translation efforts. And even if we could snap our fingers and banish overnight the biases that are among the contributors to the disappointing results, science still may not take us to the goal of improved clinical treatments that we seek. After all, we are dealing with science, not magic. But if we could muster the desire and discipline to better combat bias in research, at least we could take comfort in the fact that what we are calling science is in fact actual science, as free of bias as we can possibly make it. The two complimentary strategies described above are offered in hopes that they could help to muster that desire and discipline. If either or both were to prove beneficial, we would find ourselves in a place far preferable to the one we are in now.
